# Cryopreservation protocol for human biliary tree stem/progenitors, hepatic and pancreatic precursors

**DOI:** 10.1038/s41598-017-05858-0

**Published:** 2017-07-20

**Authors:** Lorenzo Nevi, Vincenzo Cardinale, Guido Carpino, Daniele Costantini, Sabina Di Matteo, Alfredo Cantafora, Fabio Melandro, Roberto Brunelli, Carlo Bastianelli, Camilla Aliberti, Marco Monti, Daniela Bosco, Pasquale Bartolomeo Berloco, Pierluigi Benedetti Panici, Lola Reid, Eugenio Gaudio, Domenico Alvaro

**Affiliations:** 1 Department of Medico-Surgical Sciences and Biotechnologies, Sapienza University of Rome, Rome, Italy; 2 Department of Movement, Human and Health Sciences, Division of Health Sciences, University of Rome “Foro Italico”, Rome, Italy; 3 Department of General Surgery and Organ Transplantation, Sapienza University of Rome, Rome, Italy; 4 Department of Gynaecologic-Obstetric and Urologic Sciences, Sapienza University of Rome, Rome, Italy; 5 Department of Experimental Medicine, Sapienza University of Rome, Rome, Italy; 6 Department of Cell Biology and Physiology and Program in Molecular Biology and Biotechnology, University of North Carolina School of Medicine, North Carolina, USA; 7 Department of Anatomical, Histological, Forensic Medicine and Orthopedics Sciences, Sapienza University of Rome, Rome, Italy; 8 Department of Medicine and Medical Specialties, Sapienza University of Rome, Rome, Italy

## Abstract

Human biliary tree stem/progenitor cells (hBTSCs) are being used for cell therapies of patients with liver cirrhosis. A cryopreservation method was established to optimize sourcing of hBTSCs for these clinical programs and that comprises serum-free Kubota’s Medium (KM) supplemented with 10% dimethyl sulfoxide (DMSO), 15% human serum albumin (HSA) and 0.1% hyaluronans. Cryopreserved versus freshly isolated hBTSCs were similar *in vitro* with respect to self-replication, stemness traits, and multipotency. They were able to differentiate to functional hepatocytes,cholangiocytes or pancreatic islets, yielding similar levels of secretion of albumin or of glucose-inducible levels of insulin. Cryopreserved versus freshly isolated hBTSCs were equally able to engraft into immunocompromised mice yielding cells with human-specific gene expression and human albumin levels in murine serum that were higher for cryopreserved than for freshly isolated hBTSCs. The successful cryopreservation of hBTSCs facilitates establishment of hBTSCs cell banking offering logistical advantages for clinical programs for treatment of liver diseases.

## Introduction

We have recently demonstrated the presence of cells expressing a constellation of endodermal markers in (peri)-biliary glands of intrahepatic and extrahepatic bile ducts^1–4^. These observations *in situ* in human biliary tree tissues have been complemented by *ex vivo* demonstrations that there are multiple subpopulations of biliary tree stem cells (BTSCs), all expressing PDX1, SOX17, SALL4, and CD44 and yet with distinctions in other phenotypic traits. The three most common subpopulations are ones with expression of [LGR5+/EpCAM+]; [LGR5/EpCAM-]; and a third [LGR5-/EpCAM-]. All can be isolated from the biliary epithelium and have long-term (*in vitro*) maintenance and self-renewal, and are able to proceed through different maturational lineages to adult fates that comprise hepatocytes, cholangiocytes and β-pancreatic cells^1–4^. The discovery of these cells, named human biliary tree stem/progenitor cells (hBTSCs), opens a new scenario with relevant implications in different fields including the embryology of liver, biliary epithelium and pancreas, pathophysiology of biliary tree, hepatobiliary and pancreatic carcinogenesis and, finally, regenerative medicine of liver and pancreas^1–4^. In this regard, the recent demonstration of one of subpopulations of hBTSCs found within the crypts of the gallbladders, and referred to as human gallbladder stem/progenitor cells (hGSCs)^5^, increases the possibility of a clinical use of these populations of endodermal stem/progenitor cells with multipotential and differentiative capacity (hBTSCs and hGSCs) for cell therapies of liver diseases. Importantly, these cells are easily isolatable and cultivatable and have a low or null immunogenic and oncogenic potential^6^. Given the various obstacles in cell sourcing for regenerative medicine^7^, the biliary tree could represent an ideal source of stem cells and progenitors for regenerative medicine. Indeed, we successfully transplanted freshly isolated hBTSCs in cirrhotic patients with benefits in terms of improvement of liver functions^8^.

Human tissues are difficult to obtain, and the current requirement for clinical programs, that cells be freshly isolated, hampers sourcing of cells for treatments of patients. For this reason, cryopreservation represents an obligatory step for routine uses of cell products in clinical programs of cell therapies. A number of different cryopreservation techniques have been proposed including the use of cryopreservation agents^9, 10^, a cell coating technique^11–13^, pre-conditioning techniques^14^, and gradual freezing^15, 16^. Unfortunately, with regard to cell types isolated from solid organs, like hepatic cells, a large variability in terms of cell viability and engraftment efficiency after thawing has been reported^14, 17, 18^. Terry *et al*.^14^, for example, proposed the use of purified human serum albumin as an alternative to serum in order to preserve high viability and to achieve a defined cryopreservation condition. More recently, Turner *et al*.^19^ developed an efficient strategy to preserve adhesion molecule expression during human hepatic stem cell (hHpSC) cryopreservation by using either of two serum-free, wholly defined buffers that were supplemented with hyaluronans: Crystor-10 (CS10; Biolife Solutions, Bothell, WA, USA) or Kubota’s Medium (Phoenix Songs Biologicals, Branford, CT). Kubota’s Medium (KM) was designed for endodermal stem cells and is described in more detail in the methods section. It is distinctive in having no cytokines, growth factors or hormones other than insulin and transferrin. Even though it is not isotonic, its ability as a cryopreservation buffer has proved similar to that of the Good Manufacturing Practice (GMP) approved Cryostor-10^20^. We utilized serum-free KM that was supplemented with different concentrations of human serum-derived albumin (HSA) and/or hyaluronic acid (HA) to generate conditions effective in cryopreservation of hBTSCs from adult donors under GMP like conditions.

## Results

### Viability, senescence and colony formation by cryopreserved hBTSCs

**Figure 1:**
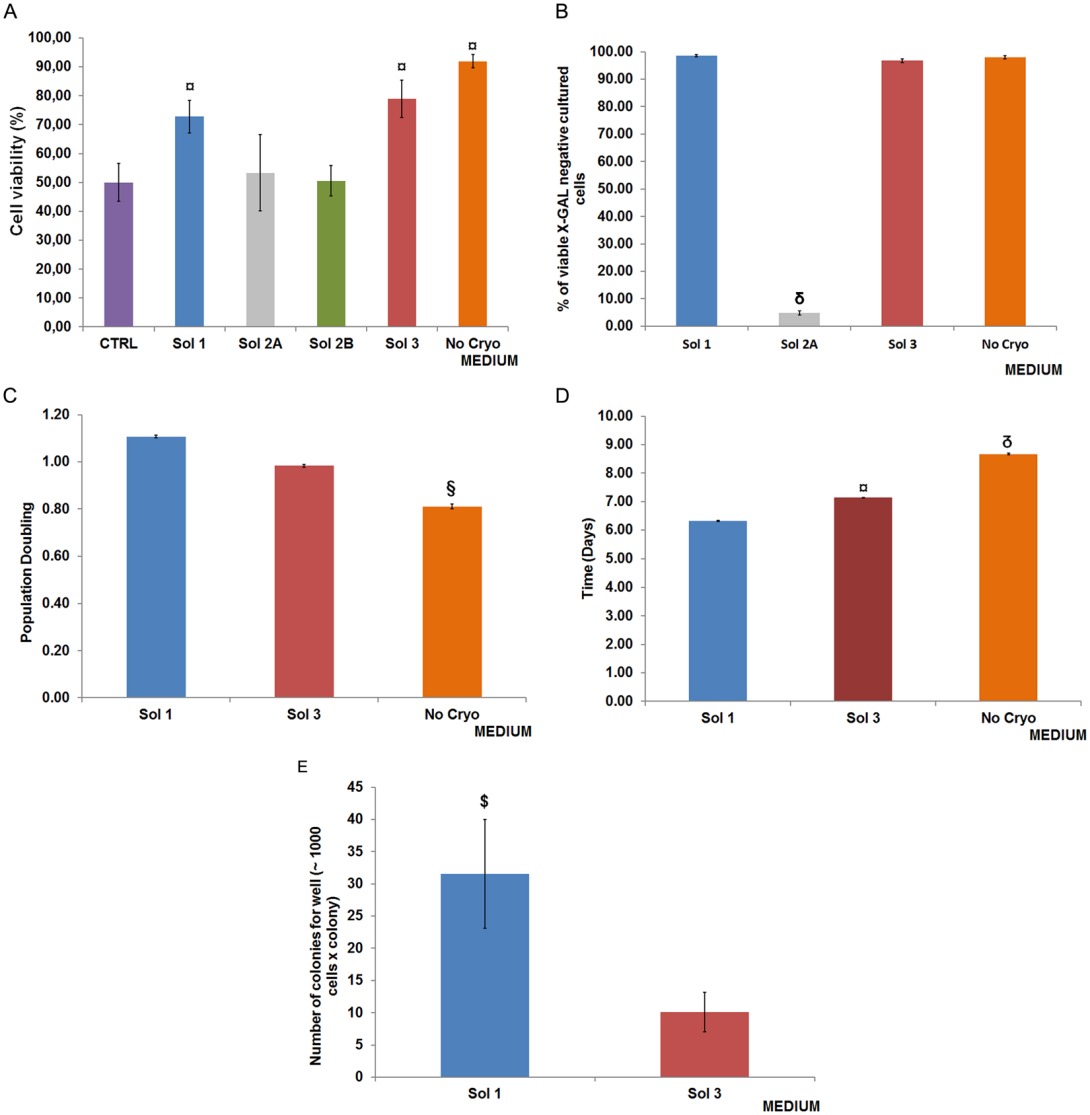
Biological cell functions after cryopreservation/thawing. (**A**) Cell viability was assessed by Trypan blue exclusion test after thawing the cells cryopreserved in different solutions (N = 9 experiments). Viability was significantly higher in solution 1 (Sol1) and Sol3 vs Sol2A, Sol2B, control solution (CTRL), and freshly isolated (No Cryo). No difference was found between Sol1 and Sol3. Data are expressed as mean ± SD of 9 experiments; ^¤^p < 0.001 Sol1 and Sol3 vs Sol2A, Sol2B, and CTRL; ^¤^p < 0.001 No Cryo vs all other Solution. Solution composition: Sol1 = Kubota Medium (KM), DMSO (10%), HSA (15%), hyaluronic acid (0.1%W/V); Sol2A = KM, hyaluronic acid (HA)(0.1%W/V), DMSO (10%); Sol2B = KM, HA (0.05% W/V), DMSO (10%); Sol3 = KM, DMSO (10%), HSA (15%); CTRL = KM, DMSO (10%), HSA (1.5%). (**B**) Cell senescence was evaluated by X-Gal test in cultures obtained from cryopreserved or freshly isolated cells (No Cryo) obtained from the same donors. Graphics show the percentage of X-Gal negative cells (non senescent cells). X-Gal negative cells exceeded 95% after cryopreservation. No difference was observed between Sol1 and Sol3, and among cryopreserved cells and fresh control cells (No Cryo). Sol2A demonstrated a massive senescence of cultured thawed cells (^δ^p < 0.0001 vs others). Data are expressed as mean ± SD of 3 experiments. (**C**) Proliferation rate expressed as population doubling (PD) week rate in cultures of hBTSCs cryopreserved in Sol1, Sol3, and freshly isolated controls (No Cryo). Cryopreserved cells (Sol1 and Sol3) demonstrated a higher PD week rate with respect non-cryopreserved cells (^§^p < 0.01). Data are expressed as mean ± SD of 8 experiments. (**D**) Population Doubling Time (PDT) appeared lower in Sol1 (with HA) than Sol3 (without HA) and freshly isolated controls (No Cryo)(^¤^p < 0.001 vs others), and in Sol3 vs freshly isolated controls (No Cryo) (p < 0.0001 vs No Cryo). Data are expressed as mean ± SD of 8 experiments. E) The number of colonies was counted at day 3 of culture. HA-coated hBTSCs and uncoated hBTSCs were compared. Graphics illustrate the number of colony formed after thawing cells cryopreserved in Sol1 and Sol3. A higher number of colonies (31.56 ± 8.43) developed in cultures from Sol1 than Sol3 (10.11 ± 3.85)(^$^p < 0.000001). Data are expressed as mean ± SD of 18 experiments.

The hBTSCs were cryopreserved in a basal control solution (10% DMSO, 1.5% HSA in KM) for 4–12 weeks and, then were thawed and seeded at a density of 10,000 cells/mL on plastic. Figure 1 and Supplementary Figure 1 show the cell viability and morphology of hBTSC cultures after 4 weeks of cryopreservation in the basal control solution. After thawing, cells were grown for a period of 30 days in Kubota’s Medium (KM). The hBTSCs were able to form cell colonies that were morphologically similar to those generated by freshly isolated cells (Supplementary Figure 1). We tested various cryopreservation buffers. All of them were comprised of serum-free KM supplemented with 10% dimethyl-sulfoxide (DMSO) and with distinctions in containing different concentrations of HSA and HA. The percent of viable cells was assessed after 4 weeks of cryopreservation and immediately after thawing (N = 9). Cells in Solution 1 (Sol1: supplemented further with 0.1% HA + 15% HSA) had an average viability of 72.78 ± 5.65%. Those in Solution 3 (Sol3: supplemented further with 15% HSA) had an average viability of 78.89 ± 6.51%. Sol1 and Sol3 yielded viabilities after thawing that were significantly higher (p < 0.001) than those in the other buffers. The average viabilities in Solution 2 A (Sol2A: supplemented with 0.1% HA) were 53.33 ± 13.23%; those in Solution 2B (Sol2B: supplemented with 0.05% HA) were 50.56 ± 5.27%, and those in the control solution (CTRL: supplemented with 1.5% HSA) were 50.00 ± 6.61%. No significant difference in cell viability was found between Sol1 and Sol3 (Fig. 1A).

We next evaluated cell senescence (X-Gal) in cultures obtained from cryopreserved or freshly isolated cells from the same donors. The number of X-Gal negative cells (not senescent) exceeded 95% after cryopreservation (Fig. 1B). No difference was observed between Sol1 (with HA; 98.57 ± 0.36; N = 9) and Sol3 (without HA; 96.72 ± 0.66; N = 9; p > 0.05), and between cryopreserved and freshly isolated cells (98.00 ± 0.53; N = 9; p > 0.05). Senescent negative cells were markedly lower in Sol2A (4.85 ± 0.80; N = 9; p < 0.0001) than other conditions. Cell population doubling (PD) in cultures confirmed the optimal maintenance of the *in vitro* functional properties of the hBTSCs cryopreserved in Sol1 and Sol3. The PD in fact, was significantly higher in Sol1 (1.11 ± 0.01) and Sol3 (0.98 ± 0.01) as compared to those that were freshly isolated (0.81 ± 0.01) (N = 8; p < 0.01) (Fig. 1C). The PD time (PDT) was significantly lower in Sol1 (with HA) than Sol3 (without HA) (6.32 ± 0.02 vs 7.14 ± 0.02 days; N = 8; p < 0.001), and in Sol3 as compared to freshly isolated cells (8.67 ± 0.03 days) (N = 8; p < 0.0001) (Fig. 1D).

Colony formation is a surrogate marker of seeding and engraftment capacity. The number of colonies, formed by 200–3,000 cells, was dramatically increased in cells cryopreserved in Sol1 (with HA, 31.56 ± 8.43, N = 18) as compared to those in Sol3 (without HA, 10.11 ± 3.85, N = 18; p < 0.000001) (Fig. 1E).

### Expression of stem cell markers and adhesion molecules in cryopreserved hBTSCs

**Figure 2:**
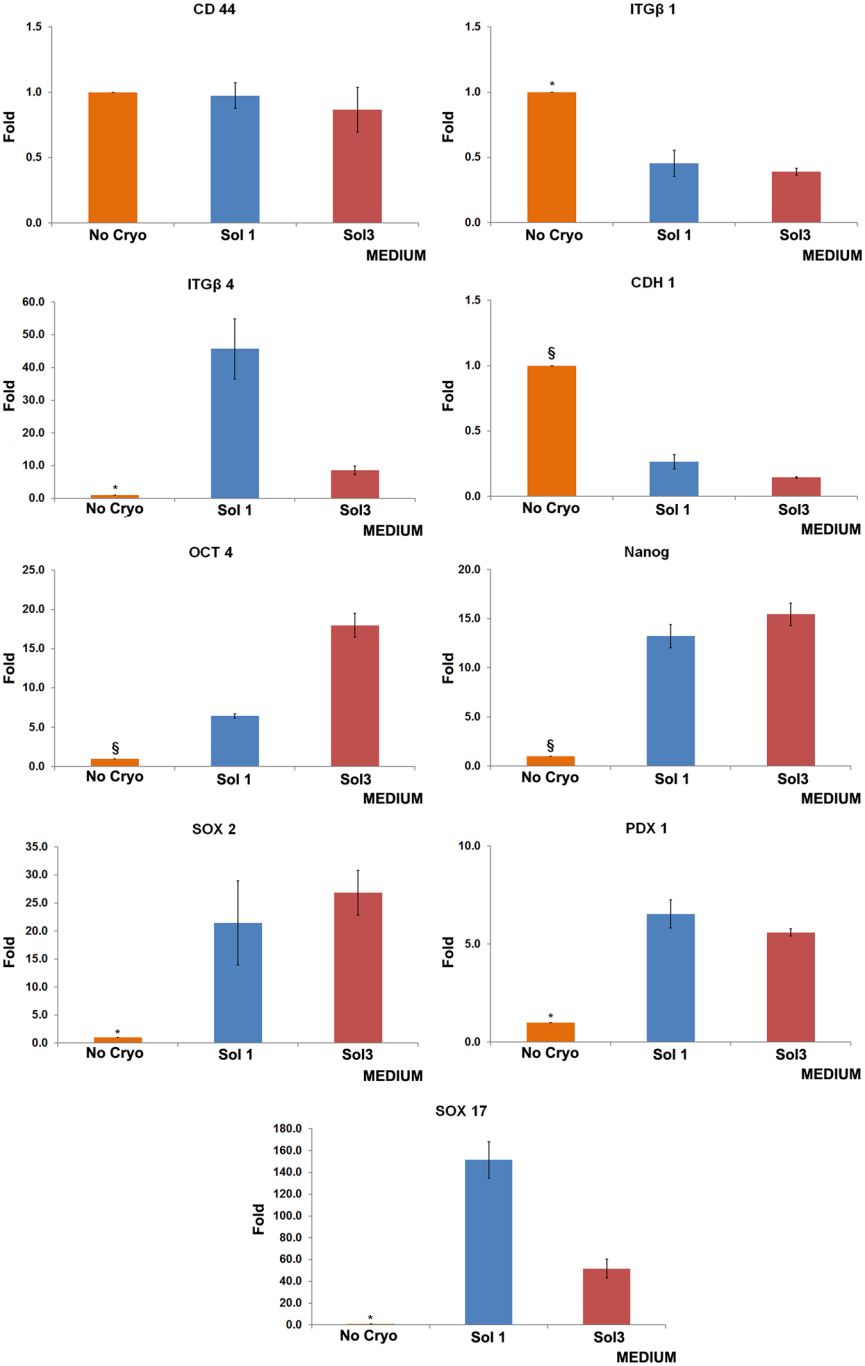
Expression of pluripotency and molecule adhesion genes in cultures from cryopreserved cells in solution 1 (Sol1), Sol3, or freshly isolated, that is not cryopreserved (No Cryo) human biliary tree stem cells (hBTSCs). Relative gene expression of SOX2. Cryopreserved hBTSCs in both Sol1 and 3 showed increased expression. Data are expressed as mean ± standard error (SE) of 9 experiments; *p < 0.05. Relative gene expression of PDX1. Cryopreserved hBTSCs in both Sol1 and 3 showed increased expression. Data are expressed as mean ± SE of 9 experiments; *p < 0.05. Relative gene expression of NANOG. Cryopreserved hBTSCs in both Sol1 and 3 showed increased expression. Data are expressed as mean ± SE of 9 experiments; ^§^p < 0.01. Relative gene expression of SOX17. Cryopreserved hBTSCs in both Sol1 and 3 showed increased expression. Data are expressed as mean ± SE of 9 experiments; *p < 0.05. Relative gene expression of OCT4. Cryopreserved hBTSCs in both Sol1 and 3 showed increased expression. Data are expressed as mean ± SE of 9 experiments; ^§^p < 0.01. Relative gene expression of CD44. Data are expressed as mean ± standard error (SE) of 6 experiments. Relative gene expression of ITGβ1. Cryopreserved hBTSCs in both Sol1 and 3 showed reduced expression. Data are expressed as mean ± SE of6 experiments; *p < 0.05. Relative gene expression of ITGβ4. Cryopreserved hBTSCs in both Sol1 and 3 showed increased expression. Data are expressed as mean ± SE of 6 experiments; *p < 0.05 No Cryo vs others. Relative gene expression of CDH1. Cryopreserved hBTSCs in both Sol1 and 3 showed reduced expression. Data are expressed as mean ± SE of 6 experiments; ^§^p < 0.01.

To evaluate whether cryopreservation affects stem cell phenotype, the expression of pivotal genes commonly expressed by endodermal stem cells was assessed. These include pluripotency genes (*OCT4*, *NANOG*, *SOX2*) and endodermal transcription factors(*SOX17*, *PDX1*). These were assessed before and after 1 month of cryopreservation. Interestingly, stem cell genes were more highly expressed in hBTSCs cryopreserved in Sol1 and Sol3 than in freshly isolated cells [*SOX2* (p < 0.05), *PDX1* (p < 0.05), *NANOG* (p < 0.01), *SOX17* (p < 0.05), and *OCT4* (p < 0.01); N = 5](Fig. 2).

As shown by Turner *et al*.^20^ engraftment after cell transplantation well correlates with the level of expression of adhesion molecules. Therefore, we analysed by RT-qPCR the gene expression of different genes encoding adhesion molecules including *CD44* (the hyaluronan receptor), *ITGB1* (integrin beta1), *ITGB4* (integrin beta 4), and *CDH1* (cadherin 1). No significant differences were found in cells subjected to different cryopreservation buffers versus freshly isolated cells in the expression of *CD44* (Fig. 2), while the expression of *ITGB1* and *CDH1* was decreased in cryopreserved cells compared to freshly isolated hBTSCs (*ITGB1*, p < 0.05; *CDH1* N = 5; p < 0.01) (Fig. 2); *ITGB4*expression increased in cryopreserved hBTSCs (p < 0.05) (Fig. 2).

### Multipotency is preserved with cryopreservation

**Figure 3:**
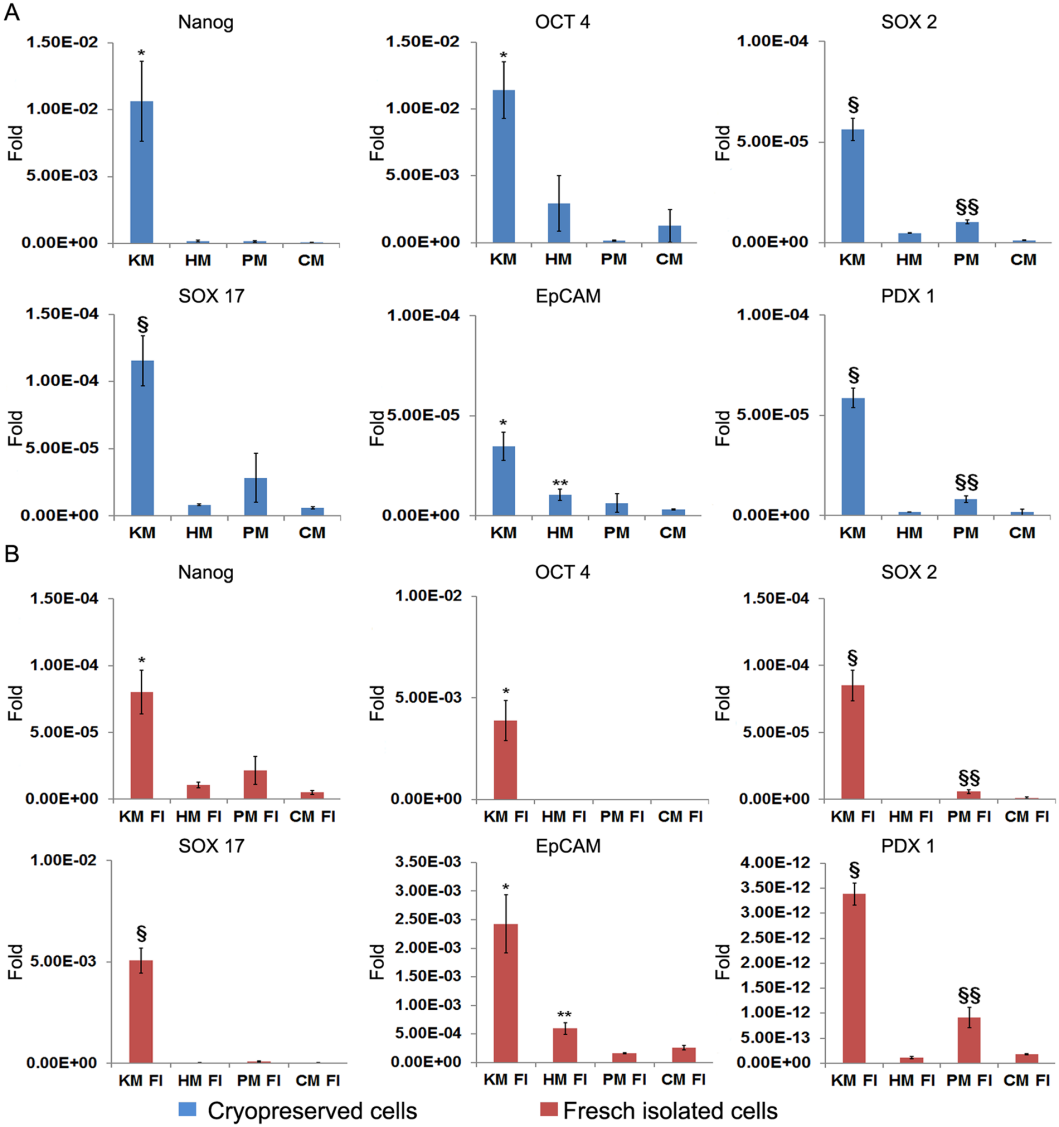
Expression of pluripotency and multipotency genes in cultures of cryopreserved or freshly isolated hBTSCs under self-renewal (KM) or hormonally defined medium for multiple endodermal mature fates (hepatocytic/HM, cholangiocytic/CM, pancreatic islets/PM). (**A**) Relative gene expression of SOX2, EpCAM, OCT4, PDX1, SOX17, SOX2 in cryopreserved hBTSCs in Sol1 and in Sol3 (not shown) under different culture conditions. Previously cryopreserved hBTSCs cultured under self-renewal conditions in Kubota’s Medium (KM) reduced the expression of pluripotency and multipotency genes when transferred in hormonally defined medium for particular endodermal mature fates (hepatocytic/HM, cholangiocytic/CM, pancreatic islets/PM). Data are expressed as mean ± SD of 3 experiments; *p < 0.05; ^§^p < 0.01; **p < 0.05 HM vs CM and PM; ^§§^p < 0.05 PM vs CM and HM. (**B**) Relative gene expression of Nanog, SOX2, EpCAM, OCT4, PDX1, SOX17, SOX2 in freshly isolated (FI) hBTSCs cultured in different defined conditions. Freshly isolated hBTSCs cultured under self-renewal conditions in Kubota’s Medium (KM) reduced the expression of pluripotency and multipotency genes when transferred in hormonally defined medium for particular endodermal mature fates (hepatocytic/HM, cholangiocytic/CM, pancreatic islets/PM). Data are expressed as mean ± SD of3 experiments; *p < 0.05; ^§^p < 0.01 **p < 0.05 HM vs CM and PM; ^§§^p < 0.05 PM vs CM and HM.

**Figure 4:**
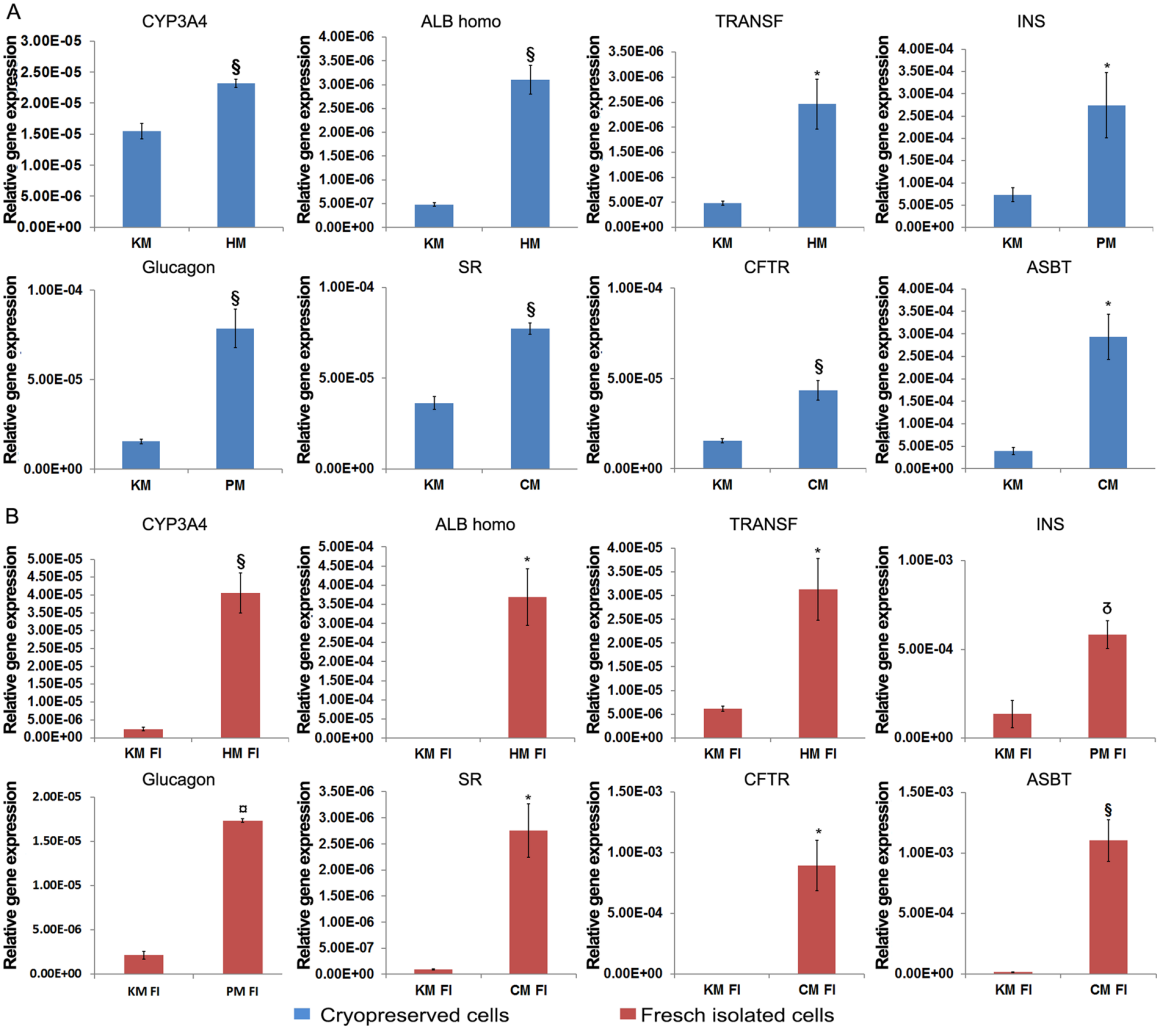
Expression of specific mature fate genes in cultures of cryopreserved or freshly isolated hBTSCs in self-renewal conditions (Kubota’s Medium-KM) or hormonally defined medium for particular endodermal mature fates (hepatoytic/HM, cholangiocytic/CM, pancreatic islets/PM). (**A**) Relative gene expression of CYP3A4, albumin (ALB), transferrin (TRANSF), insulin (INS), glucagon, Secretin Receptor (SR), CFTR, ASBT in cryopreserved hBTSCs cultured in different defined conditions. Previously cryopreserved hBTSCs cultured in self-renewal conditions in Kubota’s Medium (KM) increased the expression of specific genes associated with adult fateswhen transferred in the appropriate hormonally defined medium (hepatocytic/HM, cholangioytic/CM, pancreatic islets/PM). Data are expressed as mean ± SD of 3 experiments; *p < 0.05; ^§^p < 0.01; ¤p < 0.001; p < 0.0001. (**B**) Relative gene expression of CYP3A4, albumin (ALB), transferrin (TRANSF), insulin (INS), glucagon, Secretin Receptor (SR), CFTR, ASBT in freshly isolated hBTSCs cultured in different defined conditions. Freshly isolated hBTSCs cultured in self-renewal conditions in Kubota’s Medium (KM) increased the expression of specific genes associated with mature fates when transferred in the related hormonally defined medium (hepatocytic/HM, cholangiocytic/CM, pancreatic islets/PM). Data are expressed as mean ± SD of 3 experiments; *p < 0.05; ^§^p < 0.01; ^¤^p < 0.001; p < 0.0001.

**Figure 5:**
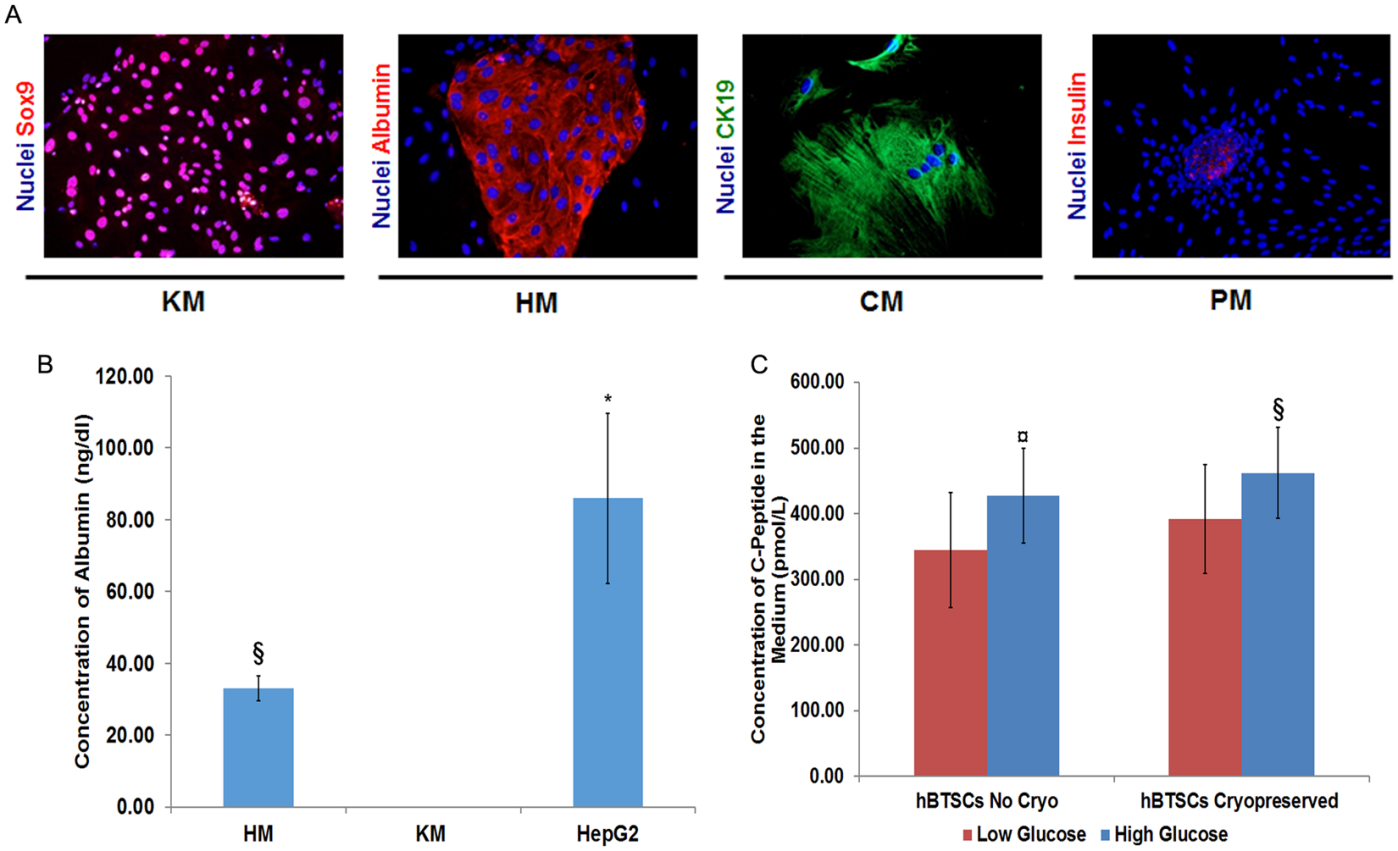
Morphological, phenotypic and functional changes induced by hormonally defined culture media compared to Kubota’s Medium/KM (basal condition) to demonstrate the effective differentiation of cryopreserved hBTSCs. (**A**) Cryopreserved hBTSCs were thawed and then cultured in media specifically tailored to induce differentiation in hepatocytes (HM), cholangiocyetes (CM) or pancreatic cells (PM). After 15 days in HM, cuboidal-shaped cells expressing albumin (hepatocyte markers) were evident (N = 5). After 15 days in CM, clusters of cells expressing CK19 appeared (N = 5). After 14 days, the monolayers in PM transition to dense balls of aggregated cells budding from the edges of the colonies and containing cells expressing insulin (Figure 10) (N = 5). Figures are representative of cultures of cells cryopreserved in Sol1 (N = 5). (**B**) The differentiation of cryopreserved hBTSCs thawed and cultured in hepatocytic medium (HM) was demonstrated by the albumin secretion with respect to control cells cultured in self-renewal conditions in Kubota’s Medium (KM) (data are expressed as mean ± SD of 6 experiments; ^§^p < 0.01 HM vs KM), that resulted lower with respect to HepG2 (*p < 0,05 HepG2 vs KM), but similar to freshly isolated cells (not shown). (**C**) In pancreatic medium (PM), both cryopreserved and freshly isolated hBTSCs acquired insulin (C-Peptide) secretion property that was regulated by glucose concentration (data are expressed as mean ± SD of 7 experiments; ^§^p < 0.01 low vs high glucose concentration, ^¤^p < 0.001 low vs high glucose concentration).

Multipotency genes are expressed in hBTSCs under self-renewal conditions and then disappear upon differentiation towards mature cells. We tested cultures of hBTSCs after cryopreservation in Sol1 (Figs 3A and 4A), Sol3 (data not shown) versus freshly isolated cells (Figs 3 B and 4B). For differentiation conditions, we used different hormonally defined media (HDM) specifically tailored to induce the differentiation of hBTSCs to mature hepatocytes (HM), cholangiocytes (CM) or pancreatic islets (PM). KM without hydrocortisone was used as a control since this medium is permissive for cell expansion and is neutral for differentiation towards both liver and for pancreas (glucocorticoids must be avoided for pancreatic differentiation). As expected, cryopreserved hBTSCs (Fig. 3A), as well as freshly isolated cells (Fig. 3B), showed decreased expression of the pluripotency genes (*NANOG*, *OCT4*, and *SOX2*) and endodermal stem cell genes (*EpCAM*, *PDX1*, and *SOX17*) after two weeks in culture in HDMs tailored for differentiation of the stem cells to hepatocytic (HM), pancreatic (PM) or biliary (CM) fates (p < 0.05). When hBTSCs (Sol1 and freshly isolated) were transferred from KM to HM for 2 weeks, significant increases in expression of mature hepatocyte-specific genes were observed including (e.g. *Albumin (Alb);* N = 5; p < 0.01 vs KM; *Transferrin (Transf);* N = 5; p < 0.05 vs KM and *Cytochrome P450 3A4 (CYP3A4);* N = 5; p < 0.01 vs KM) (Fig. 4). Similarly, when hBTSCs (Sol1 and freshly isolated) were transferred into PM or CM for 2 weeks, significant increases of pancreatic islet-specific gene expressions (Insulin (Ins), N = 5, p < 0.05; *Glucagon* N = 5, p < 0.01 PM vs KM), and of large cholangiocytes-specific gene expressions (Secretin Receptor (SR), N = 5, p < 0.01; Cystic fibrosis transmembrane conductance regulator (CFTR), N = 5, p < 0.01; Apical sodium dependent bile acid transporter (ASBT), N = 5, p < 0.05 CM vs KM) (Fig. 4) were observed. The hBTSCs in HDMs developed characteristic changes in morphology and phenotypic traits. Specifically, after 15 days in HM, cuboidal-shaped cells expressing albumin (hepatocyte markers) were observed (Fig. 5A) (N = 5); after 15 days in CM, clusters of cells expressing Cytokeratin 19 (CK19) appeared (Fig. 5A) (N = 5); while, hBTSCs in PM produced, after 14 days, dense balls of aggregated cells budding from the edges of the colonies and containing cells expressing insulin (Fig. 5A) (N = 5). No significant differences were observed between Sol1, Sol3 and freshly isolated cells (N = 5).

We then evaluated at a functional level how cryopreserved hBTSCs can be effectively differentiated *in vitro* into hepatocyte-like cells or pancreatic islet-like cells. Cryopreserved hBTSCs cultured in HM acquired the capacity to produce and secrete albumin (N = 7; p < 0.01 in HM vs KM) although at a slight lower extent with respect to HepG2 (p < 0.05) (Fig. 5B). When cultured in PM, hBTSCs acquired insulin secretion that was regulated by glucose concentration (low versus high glucose concentration; N = 7; p < 0.01 vs low glucose) (Fig. 5C).

### Effective *in vivo* engraftment of cryopreserved hBTSCs

**Figure 6:**
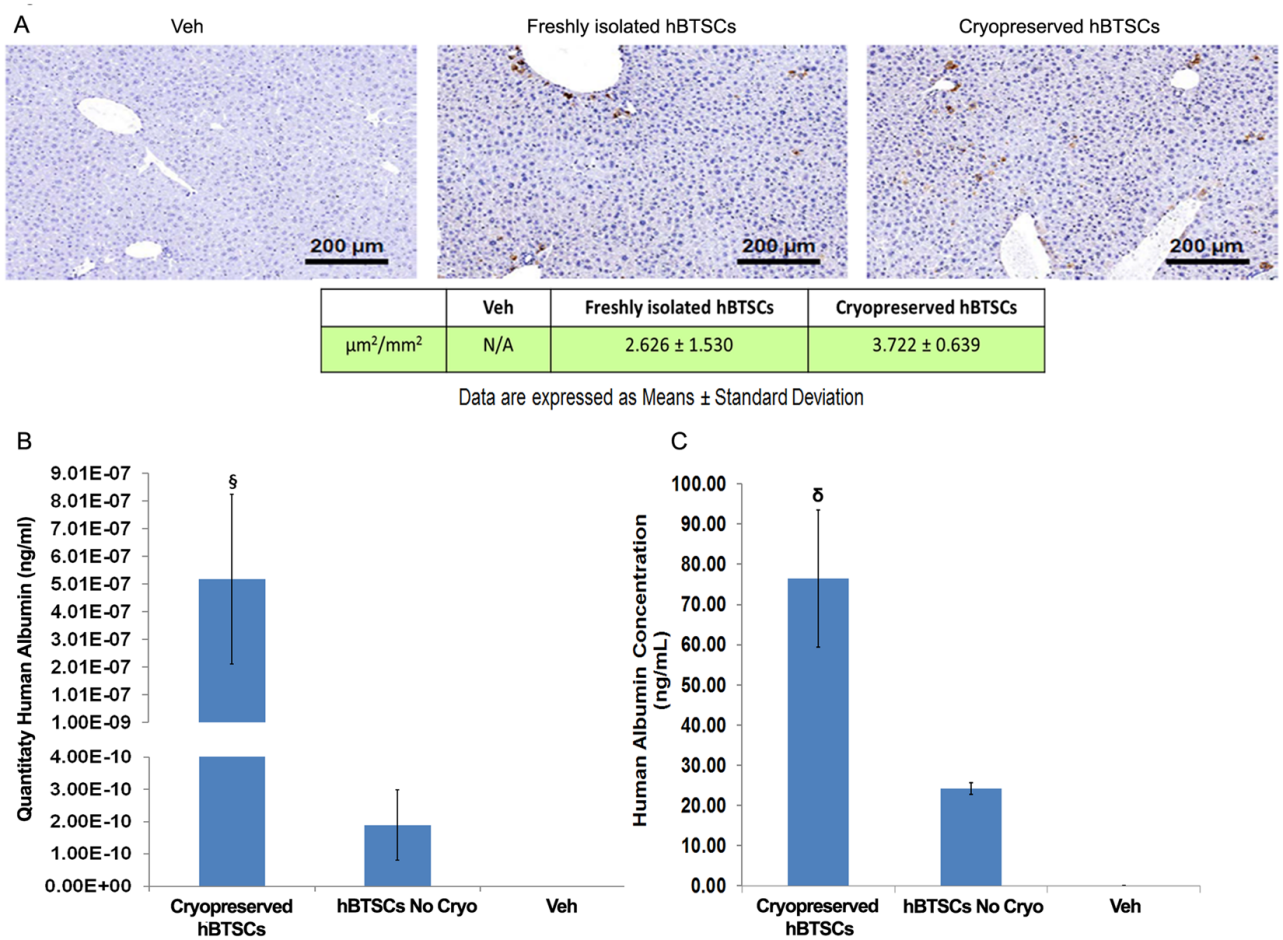
*In vivo* liver engraftment and hepatocyte differentiation of hBTSCs (cryopreserved vs freshly isolated) after intrasplenic transplantation in SCID mice. Thirty days after hBTSC injection into the spleen, livers and serum were analyzed. (**A**) Sections of livers were analyzed by immunohistochemistry utilizing anti-human mitochondria. Freshly isolated and cryopreserved hBTSCs showed similar engraftment efficiency into the murine liver parenchyma (N = 3). The expression of human mitochondria in liver parenchyma of SCID mice indicated that 2.626 ± 1.530% and 3.722 ± 0.639% of the host’s parenchyma cell mass derived from transplanted freshly isolated and cryopreserved hBTSCs respectively (data are expressed as the mean ± SD of 3 experiments). (**B**) Sections of livers were analyzed by RT-qPCR for human albumin gene expression. The gene expression of human albumin in liver parenchyma of SCID mice was higher (^§^p < 0.01) when cryopreserved hBTSCs (5.19*10^−7^ ± 3.06*10^−7^) were transplanted as compared to when freshly isolated hBTSCs (1.90*10^−10^ ± 1.09*10^−10^) were transplanted. (Data are expressed as the mean ± SD of 3 experiments). (**C**) levels of human serum albumin in the SCID mice were significantly higher (p < 0.0001) when cryopreserved hBTSCs (76.39 ± 17.04 ng/mL) were transplanted with respect to freshly isolated hBTSCs (24.13 ± 1.44 ng/mL). (Data are expressed as mean ± SD of 3 experiments).

To determine whether cryopreserved hBTSCs can effectively engraft and proliferate in the livers of immune-compromised mice, cells were transplanted into the spleen of SCID mice. The livers were then analysed 30 days after cell transplantation by immunohistochemistry with an antibody to human mitochondria as previously described^5^. As shown in Fig. 6A, cryopreserved (Sol1) hBTSCs engrafted into liver parenchyma with the same efficiency as freshly isolated cells (N = 3; p > 0.05). Indeed, the expression of human mitochondria in liver parenchyma of the SCID mice indicated that 2.626 ± 1.530% and 3.722 ± 0.639 of the host parenchymal cell mass comprised human cells derived from freshly or cryopreserved hBTSCs, respectively (Fig. 6A). To confirm the effective engraftment and differentiation of transplanted cryopreserved hBTSCs into the murine livers, we measured human albumin mRNA in the liver and human albumin (protein) in the serum. The expression of human albumin mRNA in the liver was significantly higher (N = 3; p < 0.01) in mice transplanted with cryopreserved hBTSCs (5.19*10^−7^ ± 3.06*10^−7^) than mice transplanted with freshly isolated hBTSCs (1.90*10^−10^ ± 1.09*10^−10^) (Fig. 6B). Accordingly, in the same animals, the human serum albumin levels were significantly higher (N = 3; p < 0.0001) in mice transplanted with cryopreserved hBTSCs (76.39 ± 17.04 ng/mL) than mice transplanted with freshly isolated hBTSCs (24.13 ± 1.44 ng/mL) (Fig. 6C).

## Discussion

We have established a successful cryopreservation protocol for hBTSCs comprised of serum-free Kubota’s Medium (KM) supplemented with DMSO (10%), HA (0.1%) and high concentrations of HSA (15%). The key findings leading to this conclusion arethat: 1) hBTSCs can survive and have a high viability on thawing (~80%) after 120 days of cryopreservation if subjected to this cryopreservation buffer; 2) the *in vitro* proliferation rate (population doubling times) and colony formation capacity were improved by supplementation of cryopreservation buffers with HA (0.1%); 3) hBTSCs cryopreserved in buffers containing high albumin concentrations ± HA, efficiently differentiated *in vitro* to mature fates (hepatocytes, cholangiocytes, or functional pancreatic β-cells); 4) hBTSCs cryopreserved in the buffer containing high albumin concentrations + HA effectively engrafted and differentiated *in vivo* after transplantation in SCID mice.

KM was designed for endodermal stem cells, and it is distinctive in having no cytokines, growth factors or hormones other than insulin and transferrin. Even though it is not isotonic, its ability as a cryopreservation buffer has proved similar to that of the GMP Cryostor-10^20^. Here, we utilized serum-free KM that was supplemented with different concentrations of HSA and/or HA to generate conditions effective in cryopreservation of hBTSCs from adult donors under GMP like conditions. Notably, the translation to a clinical grade GMP protocol is a ready option based on the present and previous results from a companion study^20^.

Strategies of cell cryopreservation aim to protect mechanisms of cell viability and proliferative capacity and are based on the use of isotonic buffers, antifreeze proteins (from Artic animals), antioxidants and freezing reagents such as DMSO or glycerol. The existing methods work well for hematopoietic cell subpopulations, since they inherently have extracellular matrix components that are missing cell binding domains and so the cells are able to float. Thus, their adhesion and other matrix-dependent functions are intact and not adversely affected by cryopreservation. By contrast, isolation of cells from solid organs requires enzymatic activity that dissolves the matrix enabling the cells to be dispersed into cell suspensions, making these cells vulnerable to adverse effects of cryopreservation on matrix-dependent activities^20^. Liver cells, including hepatocytes, are representative of cells from solid organs and demonstrating enormous difficulties encountered in cryopreservation^14^. In addition, cryopreservation of stem/progenitor cells has additional obstacles over those formature cells, since many additives of cryopreservation buffers, such as serum, can eliminate stemness traits and, in parallel, trigger differentiation^20^. We demonstrated previously that these obstacles can be overcome by using an isotonic medium such as Cryostor-10 or a wholly defined, serum-free stem cell medium, KM supplemented with hyaluronans, a dominant constituent of the matrix chemistry of stem cell niches^21^. In this study we were able to improve the conditions further by adding high levels of HSA (15%).We evaluated the maintenance, after cryopreservation, of key cell phenotypic properties such as viability, seeding, proliferation rate and differentiation potential^1, 19^. Firstly, we observed that cryopreservation in either serum-free buffer(Sol1 or Sol3) containing high levels of HSA (15%) resulted in a significantly better cell viability, after thawing, when compared to cells in the other buffers tested. Therefore, supplementation with high concentrations of HSA (15% compared to 1.5%) facilitates the maintenance of cell viability after thawing. Previously, Terry *et al*.^14^ had proposed that human serum albumin could represent an alternative to foetal serum assuming that the high levels of albumin contained in the serum is the major determinant of the serum cryo-protective effect; our results confirmed the role of albumin as a cryo-protective agent.

We further demonstrated that cryopreservation in solutions containing high albumin concentration ±HA protects hBTSCs from cell senescence. Cell senescence is correlated with telomere shortening during cell divisions, but, stem cells counteract senescence through high telomerase activity^22, 23^, and this has been demonstrated by Reid and associates in hepatic stem cells^22, 23^. Proliferation rates *in vitro* have been analysed by population doubling assays in which we demonstrated the preservation of the proliferation capabilities by cryopreserved hBTSCs with respect to freshly isolated cells. Seeding and proliferation are both correlated with colony formation capacity ^20^. In our study we tested whether the colony formation properties are influenced by any of the cryopreservation buffers. Expression of some adhesion molecules (e.g. *ITGB4*) was improved and that of *CD44* unaffected, whereas others were reduced (*ITGB1, CDH1*). Still proliferation in cells cryopreserved in Sol1 versus Sol3 were similar, but with dramatic increases in colony formation in those in Sol1 containing both the high albumin levels and also HA. It is noteworthy that all subpopulations of stem cells and progenitors in the liver express CD44, the receptor for HA, and that apoptosis is increased in cells that are unable to regain adhesion proteins quickly following thawing^20^. HA represents the main constituent of the liver stem cell niche^24^. Turner *et al*.^20^ observed that the use of supplementation with hyaluronans constitutes a successful option for an optimal cryopreservation of human hepatic stem/progenitor cells (hHpSC). Here we demonstrated a positive role of HA as a preconditioning agent which could favour the engraftment of cells after cryopreservation. Indeed, data obtained by RT-qPCR demonstrated that in hBTSCs cryopreserved in Sol1, the expression of adhesion molecules is partially preserved, while genes of pluripotency and endodermal stem cells are entirely preserved, as compared to expression in freshly isolated cells. These data are in agreement with the previous results by Turner *et al*.^20^. Finally and most importantly, the differentiation potential of hBTSCs was unaffected and similar to that of freshly isolated cells when cryopreserved in Sol1 or Sol3 containing high albumin concentration ±HA^1–4, 25^. Indeed, we showed *in vitro*, in media specifically tailored to induce the selective differentiation of hBTSCs to hepatocytes, cholangiocytes or pancreatic cells, that the differentiation capacities are also well preserved by our protocol of cryopreservation. They are not influenced by HA. This has also been demonstrated at a functional level by evaluating the albumin synthesis/secretion capacity of cells differentiated toward hepatocytes and insulin production, in both basal conditions and after glucose challenge, in cells differentiated toward pancreatic cells.

Finally, and most importantly, we demonstrated that hBTSCs cryopreserved in buffers containing high albumin +HA (Sol1) and transplanted into SCID mice, displayed an engraftment and differentiation efficiency even better than freshly isolated cells. The percent of human cells hosting murine liver and the synthesis and secretion of human albumin were in fact better for cryopreserved than freshly isolated hBTSCs (Sol1 vs freshly isolated). This surprising result is in keeping with observations *in vitro* in which HA improves cell engraftment and with observations *in vivo* in which cells coated with HA showed higher rates of liver engraftment, after transplantation, than freshly isolated cells. In a recent paper^26^, we demonstrated that hyaluronan coating improves liver engraftment of transplanted human biliary tree stem/progenitor cells. Therefore, it could be hypothesized that HA used during the cryopreservation process could dictate a facilitation of engraftment of the cryopreserved hBTSCs after their thawing and infusion. Thus, HA supplementation during cryopreservation could act as a preconditioning method allowing a greater number of hBTSCs to engraft within the liver parenchyma, survive, proliferate, and enhance human albumin secretion.

## Conclusions

The hBTSCs are easily isolated under GMP conditions from human tissues of donors of any age and have already been used for cell therapy of patients with advanced liver cirrhosis^27^. Given the extremely wide availability of biliary tree tissues for their isolation and given their biological characteristics, hBTSCs have enormous applicative potential for regenerative medicine of the liver and pancreas, including diabetes. In this study, hBTSCs were successfully cryopreserved without loss of crucial cell functions; this facilitates the establishment of a cell bank of hBTSCs that can be stored and used rapidly offering logistical advantages for cell therapies of liver diseases.

## Materials and Methods

### Human Tissue Sourcing

For *in vitro* experiments, human extrahepatic biliary tree, comprising common hepatic duct, bile duct, cystic duct, gallbladder, and hepato-pancreatic ampulla were obtained from organ donors from the “Paride Stefanini” Department of General Surgery and Organ Transplantation, Sapienza University of Rome, Rome, Italy. Informed consent to use tissues for research purposes was obtained from our transplant program. All samples derived from adults between the ages of 19 and 73 years. For *in vivo* experiments, hBTSCs isolated from foetal livers have been utilized. Human foetuses (16–22-week gestational age) were obtained by elective pregnancy termination from the Department of Gynaecology (Sapienza, University of Rome, Italy). Informed consent was obtained from the mother before abortion. The study was approved by the local ethics committee of the Sapienza University Hospital. Protocols received the approval of our Institutional Review Board, and processing was compliant with current Good Manufacturing Practice (cGMP like). The research protocol was reviewed and approved by the Ethic Committees of Umberto I Policlinico of Rome, Italy, and the regional committees for medical and health research ethics in South-Eastern Norway. No donor organs were obtained from executed prisoners or other institutionalised individuals.

### Tissue Processing

Tissue specimens were processed as previously described^1, 5, 6, 28–30^. In brief, tissues were digested in GMP like Serum-free Dendritic Cell Medium (CellGro # 20801–0500) supplemented with 0.1% Albunorm 20% (Octapharma # 5400454), 1 nM selenium, 300 U/ml Collagenase NB1 GMP like (Serva #17452.01), 100 U/ml Pulmozyme (Roche #18450.02), at 37° C with frequent agitation for 30–45 min. Suspensions were filtered through a 800 micron metallic mesh filter (IDEALE ACLRI9 inox stainless steel) and spun at 270 g for 10 min before resuspension. Thereafter, cell suspensions were passed consecutively through a 100 and 30 micron (µ) mesh filter; then, cell counting was done by Fast-Read 102 (BiosigmaSrl, Venice, Italy) and cell viability by the Trypan Blue assay measured (expressed as % of viable cells over total cells). Cell viability (trypan blue exclusion) was consistently higher than 95%.

### EpCAM sorting procedures

Cells were sorted for expression of Epithelial cell adhesion molecule (EpCAM) by using magnetic beads as indicated by the manufacturer (MiltenyiBiotec Inc., Germany). Briefly, the EpCAM+ cells were magnetically labelled with EpCAM MicroBeads (MiltenyiBiotec Inc., catalog #130-061-101). Then, the cell suspension was loaded onto a MACS LS Column (MiltenyiBiotec Inc., catalog #130-042-401) that was placed in the magnetic field of a MACS Separator. EpCAM+ cells were suspended in basal medium at a concentration of 300,000 cells per mL, and used as the final cell suspension.

### Cell Isolation in GMP conditions and sterility testing

To produce hBTSCs in cGMP conditions for future clinical application, gallbladders were processed following “The rules governing medicinal products in the European Union” and the European guidelines of good manufacturing practices for medicinal products for human use (EudraLex - Volume 4 Good manufacturing practice Guidelines).

### Media and solutions

All media were sterile-filtered (0.22-μm filter) and kept in the dark at 4 °C before use. RPMI-1640, the basal medium used for all the cell cultures, was obtained from GIBCO/Invitrogen (Carlsbad, CA) and the clinical grade HSA (Albunorm 20%, Cat. 5400454) was obtained from Octapharma Ltd (Switzerland). All reagents were obtained from Sigma (St. Louis, MO) unless otherwise specified. Growth factors, except those noted, were purchased from R&D Systems (Minneapolis, MN).

**Kubota’s Medium (KM)** is a serum-free medium developed for survival and expansion of endodermal stem/progenitors^31^ and subsequently shown to be successful with human hepatic stem cells^28, 29^, human biliary tree stem cells^1, 3, 4^, human pancreatic stem/progenitor cells^25^ and rodent hepatic stem cells^32^. It consists of any basal medium (here being RPMI 1640) with no copper, low calcium (0.3 mM), 10^−9^ M Selenium, 4.5 mM Nicotinamide, 0.1 nM Zinc Sulphate heptahydrate, 10^−8^ M hydrocortisone (or dexamethasone), 5 µg/mL transferrin/Fe, 5 µg/mL insulin, 10 µg/mL high density lipoprotein, 0.1% HSA, and a mixture of purified free fatty acids that are added bound to purified HSA. The detailed protocol of its preparation was first reported by Kubota and Reid^31^ and subsequently summarized in various reviews^28^. It is now available commercially through Phoenix Songs Biologicals (Branford, CT).

**For differentiation studies**, serum-free Kubota’s Medium was supplemented with calcium (final concentration 0.6 mM), copper (10^−12^ M) and 20 ng/mL basic fibroblast growth factor (bFGF) and referred to as modified Kubota’s Medium (MKM). Three different HDM have been prepared to induce selective differentiation of hBTSCs:

- **HDM for Hepatocyte differentiation (HM):** was prepared supplementing MKM with 7 μg/L glucagon, 2 g/L galactose, 1 nM triiodothyroxine 3 (T3), 10 ng/mL Oncostatin M (OSM); 10 ng/mL epidermal growth factor (EGF), 20 ng/mL hepatocyte growth factor (HGF), and 1 µM dexamethasone^4, 6^.
- **HDM for Cholangiocyte differentiation (CM):** MKM supplemented with 20 ng/mL vascular endothelial cell growth factor (VEGF) 165 and 10 ng/mL HGF^4, 6^.
- **HDM for Pancreatic islet cell differentiation (PM):** MKM without hydrocortisone, supplemented with 2% B27, 0.1 mM ascorbic acid, 0.25 µM cyclopamine, 1 µM retinoic acid; bFGF was added for the first 4 days and then replaced with 50 ng/mL exendin-4 and 20 ng/mL of HGF^4, 5^.

### Methods and Buffers for Cryopreservation

The cells were detached from the various plastic substrata to be collected and cryopreserved. Detached cell cultures were centrifuged at 270 g for 10 minutes, and 1 mL of the solution of cryopreservation was added to the cell pellets. Finally, the buffers containing the cells were transferred into Nunc vials (Unimed # 6302598). These were placed into Nalgene® Mr. Frosty® Cryo 1 °C Freezing Container (Thermo Fisher Scientific, Mexico, CAT No. 5100-0001). The method of cryopreservation used was by lowering of the temperature at 1 °C per minute to −80 °C; after 24 hours, the cells were placed in liquid nitrogen at −196 °C.

Different candidate cryopreservation buffers were tested. They were prepared on the day of use and in the amount of 10 mL each. The buffers are derivative of those established by Turner, *et al*.^19^. They all consist of Kubota’s Medium, a serum-free medium developed for endodermal stem/progenitors, supplemented with 10% DMSO; in addition, KM contains purified albumin to which is bound a mix of purified free fatty acids. In some of the buffers, additional, higher levels of clinical grade HSA (Albunorm 20% by Octapharma Ltd Switzerland, Cat. 5400454), were added. HA was obtained by SIGMA #S0780000 (St. Louis, MO).

The distinctions among the buffers are as follows:

- Sol1: HSA (15%), HA(0.1%)
- Sol2A: HA (0.1%)
- Sol2B: HA (0.05%),
- Sol3: HSA (15%),
- CTRL: HSA (1.5%),

HA was prepared using 200 mg of sodium hyaluronate suspended in 30 mL of KM.

### Cell thawing

The frozen cells in the Nunc (Unimed # 6302598) were thawed and 1 mL of KM with 20% HSA was added slowly (drop by drop). Then, the contents were transferred into a 15 mL Falcon tube; the volume was brought slowly to 5 mL with KM and then subjected to centrifugation at 270 g for 10 minutes^2^. After centrifugation the supernatant was removed, eliminating the DMSO that was used for the cryopreservation. The cell pellet was resuspended to the requisite volume for plating^1^. Analytical studies included ones assessing the cell viability of thawed cells that had been frozen with the different cryopreservation solutions, and gene expression, through the use of RT-qPCR, both of adhesion molecules (ITGB1, ITGB4, CD44, CDH1) that are markers of pluripotent stem cells and markers of endodermal stem cells (PDX1, OCT4, SOX17, SOX2, Nanog).

### Cell Cultures and clonal expansion

Unsorted and sorted EpCAM+ cells (approximately 3 × 10^5^), obtained from biliary tissue specimens, were seeded onto 3 cm diameter plastic culture dishes and kept overnight (~12 hours) in KM with 10% Foetal Bovine Serum (FBS). Thereafter, cell cultures were maintained in serum-free KM and observed for at least 2 months. For testing the clonal expansion of hBTSCs, a single cell suspension was obtained, and the cells were plated on culture plastic at a clonal seeding density (500/cm^2^)^33^ in KM, conditions under which they self-replicate every ~36–40 hrs indefinitely (especially if at low (2%) oxygen conditions).Hepatoblasts last only about 5–7 days under these conditions (they require additional factors for longer term survival and expansion). Mature epithelial cells of liver, biliary tree and pancreas do not survive beyond a week in serum-free KM.

### Cell viability

Cell viability was determined by trypan blue exclusion assay (Sigma #302 643-25G). The cells staining blue were dead; the viable cells did not stain.This dye was used 1:1 v/v with the cell solution. The cell count was carried out through the use of FAST-READ 102 (Biosigma# BSV100).Cells viability was calculated immediately after cell thawing.

### Senescence

Senescence of thawed cells was determined by the X-Gal test (Sigma #CS0030)^34^. We used a cell density of 2.6 × 10^4^ cells/cm^2^, and the cells were grown for three days before testing. The cells cryopreserved in Sol1 and Sol3, ones that had demonstrated the highest viabilities on thawing, were analysed further with the X-Gal test. The results were compared with controls: cells that had not been cryopreserved. The controls comprised cells that had been cultured, detached and then plated for the assay to imitate the process generating freshly isolated cells.

### Population Doubling

The proliferation rate wasanalysed on the same hBTSC population, seeded in 6 multi-well plates at the density of 1 × 10^4^ cell/cm^2^ and cultured for 7 days. The cell counts were performed under the following culture conditions:

- hBTSCs cryopreserved in Sol1
- hBTSCs cryopreserved in Sol3
- hBTSCs freshly isolated (not cryopreserved)

The medium was changed every three days, using serum-free KM. The mean of the cell number was calculated on three experimental samples for each condition, and cell density was expressed as the mean of cells/cm^2^ ± standard deviation (SD). Cells were detached from supports and were counted by trypan blue assay. For these experiments we used only viable cells.

The PDT was calculated in the phase exponential growth by the following equation (1)^35^:

1 $$PDT={\mathrm{log}}_{10}2x{\rm{\Delta }}T/{\mathrm{log}}_{10}({N}_{7d})-{\mathrm{log}}_{10}({N}_{1d})$$

N_7d_ is the cell number at day 7, and N_1d_ is the cell number at day 1.

To determine the PDrate, hBTSCs, were initially seeded at the density of 1 × 10^4^ cell/cm^2^ in culture medium. Three samples for each condition were used. The following equation (2)^35^ was applied:

2 $$PD={\mathrm{log}}_{10}(N)-{\mathrm{log}}_{10}({N}_{s})/\mathrm{log}10\,(2)$$

N is the harvested cell number and N_s_ is the initial plated cell number.

### Colony Counting

The hBTSC colonies began to appear between 1 and 2 weeks after plating and were easily identified by inspection at 10X with a light microscope. Any size colony was counted as one, whether large ones at >3,000 cells or small ones at <200 cells. Each well of the 8 well chamber slide was evaluated using 10X magnification for colonies and counted after 2–3 weeks of culture. Observations of colony number, size, and morphology were noted. Given that the highest viabilities on thawing were given by cells cryopreserved in buffers Sol1 and Sol3, these cells were subjected to further assays to assess their responses to freezing^19^.

### Quantitative reverse-transcription polymerase chain reaction (RT-qPCR) analysis

RNA extractions were performed on tissues from mouse liver or from hBTSC cultures. Total RNA from intrahepatic and extrahepatic biliary tree-derived cell cultures was extracted by the procedures of Chomczynski and Sacchi^36^. The protocol of reverse transcription and qPCR is reported in Supplementary Material and Methods. We have used the GAPDH and β-ACTIN as reference genes for *in vitro* and *in vivo* data respectively. More detailed method and the sequences of the primers are contained in Supplementary materials and Supplementary Table 1 respectively.

### Measurement of Albumin secretion in hBTSCs

The hBTSCs underwent a self-replication period in serum-free Kubota’s Medium (KM) after plating on culture plastic. Cells were seeded at the density of 3.8 × 10^5^ cells/cm^2^ in KM. The medium was changed every 3 days. After 1 week of culturing in KM, the cultures were subjected either to KM (controls) or to an HDM tailored for hepatocytes. The albumin secretion experiment was performed after a further 2 weeks of culturing. For the entire period of the assay, the cells were not passaged.

Cell culture medium collected over 24 hours was analysed in triplicate by the human albumin-specific ELISA kit (Albumin Human ELISA Kit, Abcam, Cambridge, UK, catalog# ab108788). Medium was collected and stored at −20 °C. Values are expressed as micrograms per million cells per millilitre culture medium. The evaluation of the human albumin secretion in the supernatant medium has been also performed in HepG2 cells purchased commercially from Lonza (Basel, Switzerland), a well differentiated human hepatocellular carcinoma cell line, utilized as a positive control.

### Measurement of C-peptide secretion in hBTSCs

The hBTSCs underwent a self-replication period in serum-free KM after plating on culture plastic. Cells were seeded at the density of 5.2 × 10^5^ cell/cm^2^ in KM. The medium was changed every 3 days. After 1 week of culturing in KM, the cultures were subjected either to KM (controls) or to an HDM tailored for differentiation of the stem cells to pancreatic islets. The glucose challenge experiment was performed after further 2 weeks of culturing. For the entire period of the assay, the cells were not passaged.

Cells were washed three times with Dulbecco’s Phosphate Buffered Saline(DPBS, GIBCO, Catalog# 14190144). Afterwards cells were incubated for 2 hours with Connaught Medical Research Laboratories medium (CMRL) with 5.5 mM glucose and antibiotics; CMRL is a chemically defined, protein-free medium with higher levels of nucleosides and vitamins and found useful for human and primate cells. The incubation medium was collected and stored at −20 °C. Cells were again gently washed three times with DBPS and then incubated for 2 hours in glucose-free CMRL supplemented with 28 mM glucose and antibiotics. Again, medium from each well was collected and stored at −20 °C. Cells were counted using Trypan Blue assays. Samples from cultures at 5.5 mM versus 28 mM glucose were used for assays of C-peptide synthesis. The human C-peptide content in the medium was measured by an ELISA kit (R&D, Ref DICP00) and normalized to the cell number of each sample. The amount of C-peptide generated in response to the high-glucose challenge was divided by the amount generated by the low-glucose challenge to yield the mean C-peptide secretion index. The stimulation index of C-peptide secretion is calculated as the ratio between C-peptide secreted in the medium under high glucose concentration and C-peptide secreted under basal (low) glucose concentration; C-peptide concentration in the medium was quantified by ELISA in the same cell sample and during a fixed time period (2 h).

### Cell transplantation in SCID mice

All animal experiments have been carried out in accordance with the EU Directive 2010/63/EU for animal experiments and with Sapienza institutional guidelines. The animal experimental protocol was reviewed and approved by the Ethic Committee of Sapienza University of Rome and Umberto I University Hospital of Rome (Prot. 541).SCID mice (T/SOPF NOD.CB17 PRKDC/J) (N = 4) were male, 4-week old animals and were used as the hosts for transplantation of human cells. Animals were sedated with an anaesthetic drug (2, 2, 2-Tribromoethanol). Thereafter, freshly isolated or cryopreserved and thawed 2 × 10^6^ hBTSCs were suspended in 100 µl saline and injected into the liver via the spleen. Sham controls were infused only with 100 µl saline. All the animals were closely monitored until recovery, and were allowed free access to food and water. All the animal protocols complied with our institutional guidelines. No mortality occurred. At 30 days after cell transplantation, mice were sacrificed, and the livers removed for further analyses. Liver samples were placed in Trizol Reagent for gene analyses or in 4% formalin for pathologic and immunohistochemistry analyses. Blood samples were collected from the heart, centrifuged and serum samples stored at −20 °C for quantification of human albumin by ELISA (ABCAM #ad108788).

### Light Microscopy (LM), Immunohistochemistry (IHC) and Immunofluorescence (IF)

Specimens were fixed in 10% buffered formalin for 2–4 hours, embedded in low-temperature-fusion paraffin (55–57 °C), and 3–4 µm sections were stained with haematoxylin-eosin and Sirius red/Fast green, according to standard protocols. For IHC, endogenous peroxidase activity was blocked by 30 min incubation in methanolic hydrogen peroxide (2.5%). Antigens were retrieved, as indicated by the vendor, by applying Proteinase K (Dako, code Sol3020) for 10 min at room temperature. Sections were then incubated overnight at 4 °C with primary antibodies **(**Supplementary Table 2 **)**. Samples were rinsed twice with PBS for 5 min, incubated for 20 min at room temperature with secondary biotinylated antibody (LSAB+ System-HRP, Dako, code K0690; Glostrup, Denmark) and then with Streptavidin-HRP (LSAB+ System-HRP, Dako, code K0690). Diaminobenzidine (Dako) was used as substrate, and sections were counterstained with haematoxylin. For immunofluorescence on cell culture, slides chambers were fixed in acetone for 10 min at room temperature and then rinsed with PBS-Tween 20. Non-specific protein binding was blocked by 5% normal goat serum. Fixed cells were incubated with primary antibodies. Then, cells were washed and incubated for 1 h with labelled isotype-specific secondary antibodies (anti-mouse AlexaFluor-546, anti-mouse Alexafluor-488, anti-rabbit Alexafluor-488, anti-goat AlexaFluor-546, Invitrogen, Life Technologies Ltd, Paisley, UK) and counterstained with 4,6-diamidino-2-phenylindole (DAPI) for visualization of cell nuclei. For all immunoreactions, negative controls (the primary antibody was replaced with pre-immune serum) were also included. Sections/Cultures were examined in a coded fashion by Leica Microsystems DM 4500 B Light and Fluorescence Microscopy (Weltzlar, Germany) equipped with a JenoptikProg Res C10 Plus Videocam (Jena, Germany). IF staining was also analysed by Confocal Microscopy (Leica TCS-SP2). LM, IHC and IF observations were processed with an Image Analysis System (IAS - Delta Sistemi, Roma- Italy) and were independently performed by two pathologists in a blind fashion. A list of positive and negative controls is included in Supplementary Table 3.

All counts have been performed in six non-overlapping fields (magnification x20) for each slide; at least 3 different slides have been taken from each specimen. For IHC/IF staining, the number of positive cells was counted in a random, blinded fashion in six non-overlapping fields (magnification x20) for each slide/culture, and the data are expressed as % positive cells.

### Statistical Analysis

Data were expressed as mean ± SD. Statistical analyses were performed by SPSS statistical software (SPSS Inc. Chicago IL, USA). Differences between groups for non-normal distribution parameters were tested by Mann–Whitney U tests. Statistical significance was set to a p-value < 0.05.

### Disclosure of Potential Conflicts of Interest

The authors have declared that no conflict of interest exists. An exception exist for LMR who has an equity position in a biotech company, *Phoenix Songs Biologics*, that has a license for the non-clinical use of some of the IP from her lab.VC, GC, LMR, EG, and DA are inventors of international patents related to hBTSCs.

## Electronic supplementary material

Supplementary Material and Methods

Supplementary Figure 1

Supplementary Table 1

Supplementary Table 2

Supplementary Table 3
