# Supplementary material for: Cryopreservation protocol for human biliary tree stem/progenitors, hepatic and pancreatic precursors
Source: Sci Rep. 2017 Jul 20;7:6080. doi: 10.1038/s41598-017-05858-0 (PMC5519713; doi:10.1038/s41598-017-05858-0)
Supplement: Supplementary file 1 — Supplementary Material and Methods [file 41598_2017_5858_MOESM1_ESM.pdf]

Cryopreservation protocol for human biliary tree stem/progenitors, hepatic and pancreatic precursors

Lorenzo Nevi[a,1], Vincenzo Cardinale[a,1], Guido Carpino[b], Daniele Costantini[a], Sabina Di Matteo[a], Alfredo Cantafora[a], Fabio Melandro[c], Roberto Brunelli[d], Carlo Bastianelli[d], Camilla Aliberti[d], Marco Monti[d], Daniela Bosco[e], Pasquale Bartolomeo Berloco[c], Pierluigi Benedetti Panici[d], Lola Reid[f], Eugenio Gaudio[g]*and Domenico Alvaro[h,]*

SUPPLEMENTARY MATERIAL AND METHODS

Protocol for RT-qPCR. This protocol describes the detailed experimental procedure for real-time RT-qPCR using SYBR Green. The procedure begins with reverse transcription of total RNA. The cDNA is then used as template for real-time PCR with gene specific primers.

Oligonucleotide Primers. Gene specific primers are retrieved from Probefinder assay design software (https://lifescience.roche.com/en_it/brands/universal-probe-library.html). These primers are ordered from the TIB BIOMOL (service@tibmolbiol.it). All the primers are desalted and both UV absorbance and capillary electrophoresis are used to assess the quality of primer synthesis. Their characteristics are reported by the Supplementary Table 1.

Reverse Transcription. Reverse Transcription is carried out with the Thermo Cycle (QANTARUS #7013000) as described here for each 0.2 mL thin-walled:

1. Add 1 µL of Oligo dT (Invitrogen #18418012),
2. Add 9.5 µL of total RNA sample,
3. Add 1 µL of dNTP (Roche #11581295001),
4. Add 1 µL of distilled RNA DNA-free H_2O (GIBCO #10977-049),
5. Spin for 10 seconds,
6. Insert samples into Termo Cycle and started program PRE-RT Reaction (from 37°C to 65°C, after 5 minutes down temperature to 10°C),
7. Spin for 10 seconds,
8. Add 4 µL of First Strand Buffer 5X (Invitrogen #1150236),
9. Add 2 µL of DTT 0.1M,
10. Add 0.5 µL of RNA inhibitor (Roche # 03335399001),
11. Incubate at 37°C for 2 minutes,
12. Add 1 µL of M-MLV (Invitrogen # 28025-013).
13. Insert samples into Termo Cycle and start the RT Reaction thermal program (from 10°C to 37°C, after 50 minutes rise the temperature to 70°C for 15 minutes, then down temperature to 10°C).

Real-time PCR. Pipet in each 0.2 mL thin-walled tube with the optical cap up to the total volume of 25 µL:

1. 12.5 µl of Mix SYBR Green (Agilent # 600828, Rox included);
2. 0.3 µl of diluited ROX (1:2000 using distilled RNA DNA-free H_2O);
3. 0.3 µl of Primer Forward (TIB BIOMOL);
4. 0.3 µl of Primer Reverse (TIB BIOMOL);
5. 10.6 µl of distilled RNA DNA-free H_2O;
6. 1 µl of sample.

Gene expression were determined by Real-Time PCR with a MX3000P instrument (Agilent, La Jolla, CA, USA) using the averaged cycle threshold (Ct) automatically computed by the built-in software from three replicas of each sample. All real-time PCR amplifications were conducted with the cycling program: 10 min at 95 °C followed by 40 cycles (30 secs at 95 °C, 30 secs at 58 °C, 30 secs at 72 °C). The fluorescence detection was performed during the extension step of each cycle.

The following genes of interest (GOI) were measured: CD44, ITGβ1, ITGβ4, CDH1, OCT4, NANOG, SOX2, PDX1, SOX17, EpCAM, CYP3A4, ALB homo, TRANSF, INS, GLUCAGON, SR, CFTR, ASBT. All expression levels were normalized to the expression of *in vitro* GAPDH and *in vivo* β-ACTIN housekeeping genes. GAPDH was used as reference gene for *in vitro* data reported in Figures 2, 3, and 4, while the β-ACTIN was used as the reference gene for *in vivo* experiments shown in Figure 6B
