# Supplementary material for: Cryopreservation protocol for human biliary tree stem/progenitors, hepatic and pancreatic precursors
Source: Sci Rep. 2017 Jul 20;7:6080. doi: 10.1038/s41598-017-05858-0 (PMC5519713; doi:10.1038/s41598-017-05858-0)
Supplement: Supplementary file 2 — Supplementary Figure 1 [file 41598_2017_5858_MOESM2_ESM.pdf]

Cryopreservation protocol for human biliary tree stem/progenitors, hepatic and pancreatic precursors

Lorenzo Nevi[a,1], Vincenzo Cardinale[a,1], Guido Carpino[b], Daniele Costantini[a], Sabina Di Matteo[a], Alfredo Cantafora[a], Fabio Melandro[c], Roberto Brunelli[d], Carlo Bastianelli[d], Camilla Aliberti[d], Marco Monti[d], Daniela Bosco[e], Pasquale Bartolomeo Berloco[c], Pierluigi Benedetti Panici[d], Lola Reid[f], Eugenio Gaudio[g]*and Domenico Alvaro[h,]*

Supplementary Data

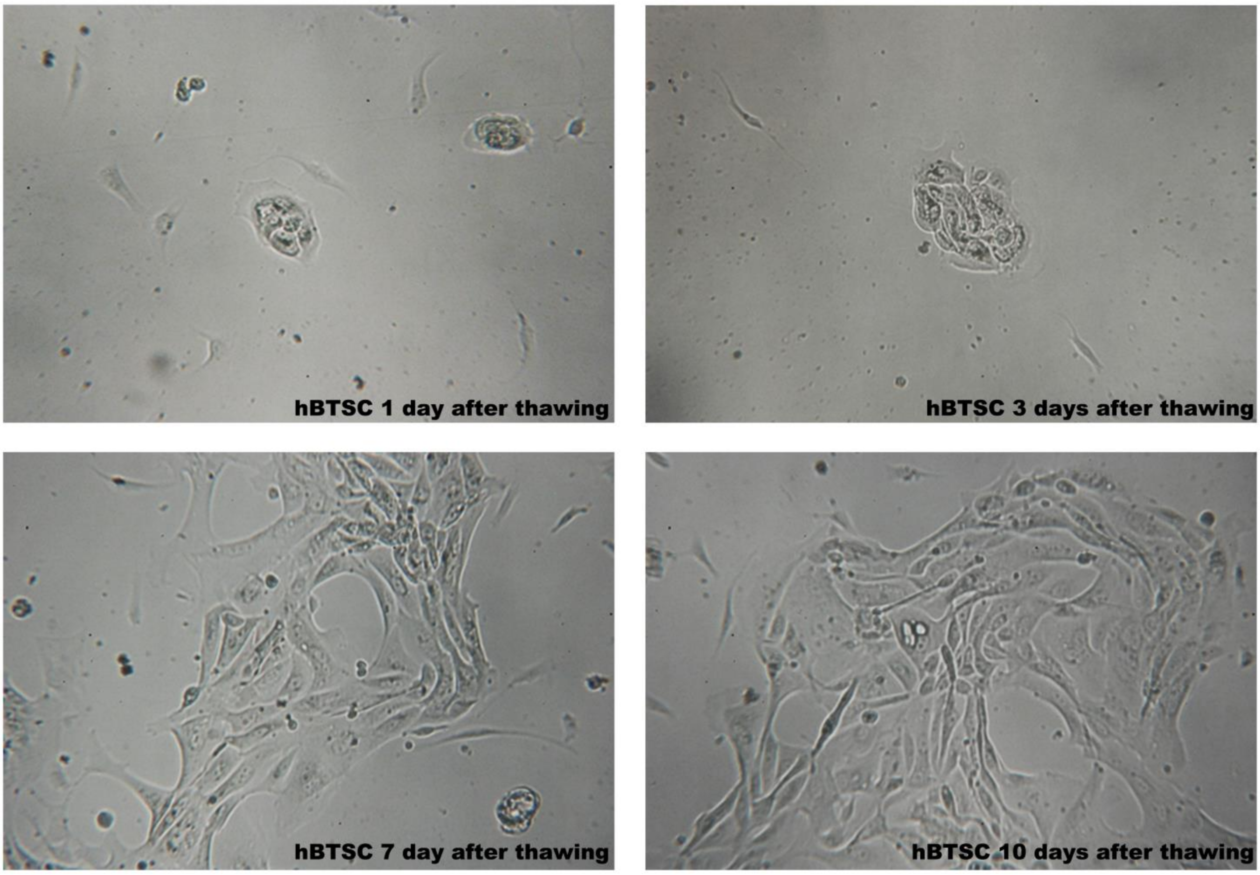

Supplementary Figure 1. Single cell clonogenity

Contrast phase imagines (Magnifications 10x) of a single colony at different culture times. A) Day 1, B) Day 3, C) Day 7, D) Day 10.
