# Supplementary material for: Cryopreservation protocol for human biliary tree stem/progenitors, hepatic and pancreatic precursors
Source: Sci Rep. 2017 Jul 20;7:6080. doi: 10.1038/s41598-017-05858-0 (PMC5519713; doi:10.1038/s41598-017-05858-0)
Supplement: Supplementary file 3 — Supplementary Table 1 [file 41598_2017_5858_MOESM3_ESM.pdf]

Cryopreservation protocol for human biliary tree stem/progenitors, hepatic and pancreatic precursors

Lorenzo Nevi[a,1], Vincenzo Cardinale[a,1], Guido Carpino[b], Daniele Costantini[a], Sabina Di Matteo[a], Alfredo Cantafora[a], Fabio Melandro[c], Roberto Brunelli[d], Carlo Bastianelli[d], Camilla Aliberti[d], Marco Monti[d], Daniela Bosco[e], Pasquale Bartolomeo Berloco[c], Pierluigi Benedetti Panici[d], Lola Reid[f], Eugenio Gaudio[g]*and Domenico Alvaro[h,]*

Supplementary Table 1. End-point determination details

The following genes of interest (GOI) were amplified using the primer pairs reported for each of them. The ratio of concentrations of GOI and the reference genes, namely, GAPDH for CDH1, CD44, ITGB1/4, OCT4, NANOG, SOX2, PDX1, SOX17,EpCAM, CYP3A4, TRANSFERRIN, INS, GLUCAGON, SR, CFTR, ASBT, and beta-actin (β-ACT) for human and murine albumin, was assumed to be the GOI relative expression.

Gene	Id. Sequence	Primers (5' – 3')
GAPDH	NM_002046.3	AAGGTGAAGGTCGGAGTCAA
		AATGAAGGGGTCATTGATGG
CD 44 Hyaluronan receptor	NM_000610.3	TGCCGCTTTGCAGGTGTAT
		GGCCTCCGTCCGAGAGA
ITGB 1 Integrin β 1	NM_002211.3	CAAAGGAACAGCAGAGAAGC
		ATTGAGTAAGACAGGTCCATAAGG
ITGB 4 Integrin β 4	NM_000213.3	CTGTGTTGCACGAGGGACATT
		AAGGCTGACTCGGTGGAGAA
CDH 1 E-Cadherin	NM_004360.3	TCACAGTCACTGACACCAACGGA
		GGCACCTGACCCTTGTACGT
OCT4	NM_002701	TCGAGAACCGAGTGAGAGG
		GAACCACACTCGGACCACA
Nanog	NM_000615	AGATGCCTCACACGGAGACT
		GGTCCTCTCCTCCTCCGTTCG
SOX2	NM_003106	TCGAGAACCGAGTGAGAGG

		GCAAAGCTCCTACCGTACCA
PDX1	NM_000209	CATTGGAAGGCTCCCTAACA
		TTCCACTGGCATCAATTTCA
SOX17	NM _022454	AAGATGCTGGGCAAGTCGTGG
		CTTGTAGTTGGGGTGGTCCTG
EpCAM	NM_002354.2	CCATGTGCTGGTGTGTGA
		TGTGTTTTAGTTCAATGATGATCCA
CYP3A4	NM_017460	AAGTCGCCTCGAAGATACACA
		AAGGAGAGAACACTGCTCGTG
TRANSFERRIN	NM_001063	CCTCCTACCTTGATTGCATCAG
		TTTTGACCCATAGAACTCTGCC
INS	NM_000000	GCAGCCTTTGTGGAACCAACAC
		CCCCGCACACTAGGTAGAGA
GLUCAGON	NM_002054	GACAAGCGCCATTCACAGG
		TGACGTTTGGCAATGTTATTCCT
SR	NM_002980.2	CTCAATGGGGAGGTGCAGCTGGA
		CTCTCAGATGATGCTGGTCCTG
CFTR	NM_000492	AAAAGGCCAGCGTTGTCTCC
		TGAAGCCAGCTCTCTATCCCA
ASBT	NM_000000	TGTGTTGGCTTCCTCTGTCAG
		GGCAGCATCCTATAATGAGCAC
Mus musculus β-actin	NM_007393.5	GGATGCAGAAGGAGATTACTGC
		CCACCGATCCACACAGAGTA
Human albumin	NM_000477.5	AGAGGTCTCAAGAAACCTAGGAAA
		GGTTCAGGACCACGGATAGA
Mus musculus albumin	NM_009654.3	CGAGAAGCTTGGAGAATATGGA
		CTTGGTGCCCACTCTTCCTA
