# Supplementary material for: Cryopreservation protocol for human biliary tree stem/progenitors, hepatic and pancreatic precursors
Source: Sci Rep. 2017 Jul 20;7:6080. doi: 10.1038/s41598-017-05858-0 (PMC5519713; doi:10.1038/s41598-017-05858-0)
Supplement: Supplementary file 4 — Supplementary Table 2 [file 41598_2017_5858_MOESM4_ESM.pdf]

Cryopreservation protocol for human biliary tree stem/progenitors, hepatic and pancreatic precursors

Lorenzo Nevi[a,1], Vincenzo Cardinale[a,1], Guido Carpino[b], Daniele Costantini[a], Sabina Di Matteo[a], Alfredo Cantafora[a], Fabio Melandro[c], Roberto Brunelli[d], Carlo Bastianelli[d], Camilla Aliberti[d], Marco Monti[d], Daniela Bosco[e], Pasquale Bartolomeo Berloco[c], Pierluigi Benedetti Panici[d], Lola Reid[f], Eugenio Gaudio[g]*and Domenico Alvaro[h,]*

Supplementary Table 2. List of used antibodies and their application(s)

**Name**	**Host / isotype**	**Source**	**Catalog#**	**Dilution**	**Application**
SOX9	Rabbit IgG	Millipore	AB5809	1:200	IHC/IF
Insulin	Guinea pig IgG	DAKO	IS002	1:100	IHC/IF
Human Albumin	Rabbit IgG	Abcam	Ab2406	1:200	IHC/IF
Cytokeratin 19	Mouse IgG1	DAKO	M0888	1:100	IHC/IF
Anti-Human Mitochondria	Mouse IgG1	Chemicon	MAB1273	1:200	IHC
