# Supplementary material for: Cryopreservation protocol for human biliary tree stem/progenitors, hepatic and pancreatic precursors
Source: Sci Rep. 2017 Jul 20;7:6080. doi: 10.1038/s41598-017-05858-0 (PMC5519713; doi:10.1038/s41598-017-05858-0)
Supplement: Supplementary file 5 — Supplementary Table 3 [file 41598_2017_5858_MOESM5_ESM.pdf]

Cryopreservation protocol for human biliary tree stem/progenitors, hepatic and pancreatic precursors

Lorenzo Nevi[a,1], Vincenzo Cardinale[a,1], Guido Carpino[b], Daniele Costantini[a], Sabina Di Matteo[a], Alfredo Cantafora[a], Fabio Melandro[c], Roberto Brunelli[d], Carlo Bastianelli[d], Camilla Aliberti[d], Marco Monti[d], Daniela Bosco[e], Pasquale Bartolomeo Berloco[c], Pierluigi Benedetti Panici[d], Lola Reid[f], Eugenio Gaudio[g]*and Domenico Alvaro[h,]*

Supplementary Table 3. Positive and Negative Controls

ANTIGEN	METHODS	POSITIVE CONTROL	NEGATIVE CONTROL
Albumin	ELISA	Mature human hepatocytes (human blood serum)	Mature cholangiocytes (hBTSCs no differentiate)
C-Peptide	ELISA	Mature human β pancreatic cells	(hBTSCs no differentiate)
human mithocondria	IHC/IF	Human liver	Mouse Liver
Hep-Par1	IHC/IF	Human liver	Mouse Liver
human Albumin	IHC/IF	Human liver	Mouse Liver
